# Probiotics Alleviated Nonalcoholic Fatty Liver Disease in High-Fat Diet-Fed Rats via Gut Microbiota/FXR/FGF15 Signaling Pathway

**DOI:** 10.1155/2021/2264737

**Published:** 2021-08-17

**Authors:** Minmin Luo, Junbin Yan, Liyan Wu, Jinting Wu, Zheng Chen, Jianping Jiang, Zhiyun Chen, Beihui He

**Affiliations:** ^1^Key Laboratory of Integrative Chinese and Western Medicine for the Diagnosis and Treatment of Circulatory Diseases of Zhejiang Province, The First Affiliated Hospital of Zhejiang Chinese Medical University, Hangzhou, 310006 Zhejiang, China; ^2^Department of Gastroenterology, Tongde Hospital of Zhejiang Province, Hangzhou, 310012 Zhejiang, China; ^3^Department of Pharmacy, School of Medicine, Zhejiang University City College, Hangzhou, 310015 Zhejiang, China; ^4^Zhejiang You-du Biotech Limited Company, Quzhou, 324000 Zhejiang, China

## Abstract

Gut microbiota (GM) dysbiosis and bile acid (BA) metabolism disorder play an important role in the pathogenesis of nonalcoholic fatty liver disease (NAFLD). Probiotics had a beneficial effect on NAFLD, but further study is needed to explore probiotics as a potential therapeutic agent to NAFLD. The aim of this study was to investigate the regulatory effect of probiotics on gut microbiota in NAFLD rats and to explore the possible mechanism of probiotics regulating the bile acid receptor farnesoid X receptor/growth factor 15 (FXR/FGF15) signaling pathway in rats. We established a rat model of NAFLD fed with a high-fat diet (HFD) for 14 weeks, which was given different interventions (312 mg/kg/day probiotics or 10 mg/kg/day atorvastatin) from the 7^th^ week. Serum lipids and total bile acids (TBA) were biochemically determined; hepatic steatosis and lipid accumulation were evaluated with HE staining. The expression levels of FXR, FGF15 mRNA, and protein in rat liver were detected. 16S rDNA was used to detect the changes of gut microbiota in rats. Compared with the HFD group, probiotics and atorvastatin significantly reduced serum lipids and TBA levels. And probiotics increased dramatically the expression of FXR, FGF15 mRNA, and protein in the liver. But there were no significant changes in the atorvastatin group. Probiotics and atorvastatin can upregulate the diversity of gut microbiota and downregulate the abundance of pathogenic bacteria in NAFLD model rats. In summary, probiotics alleviated NAFLD in HFD rats via the gut microbiota/FXR/FGF15 signaling pathway.

## 1. Introduction

Nonalcoholic liver disease (NAFLD) is the most common cause of chronic liver disease worldwide, which is predicted to become the most frequent indication for liver transplantation in the next decade [1]. NAFLD is confined to liver-related morbidity and mortality, but now, more and more evidence shows that NAFLD is a multifactorial disease. It is strongly associated with dyslipidemia, obesity, hypertension, and diabetes [2, 3]. However, the pathogenesis of NAFLD is not totally clear, and it lacks effective pharmacological treatments.

Recently, **b**ile acid metabolism plays an essential role in regulating the absorption of food lipids and cholesterol metabolism and also participates in the balance of glucose and lipid metabolism, mainly by regulating farnesoid X receptor (FXR) and then inducing the expression of fibroblast growth factor 15 (FGF15) [4, 5]. Many studies have revealed the role of gut microbiota in the pathophysiology of NAFLD, including the dysbiosis of gut microbiota composition and abundance, which leads to the destruction of intestinal endothelial barrier function and can further induce bacterial translocation and liver inflammation [6, 7]. Therefore, gut microbiota and bile acids play a key role in NAFLD and may be potential therapeutic targets.

Probiotics are live microorganisms present in cultured dairy products, which play a fundamentally important role in health and disease [8–10]. A study has shown that probiotics can reduce liver injury and improve liver function in patients with NAFLD [11]. Probiotics can regulate gut microbiota, enhance intestinal barrier function, regulate the immune system [12, 13], and improve liver lipid metabolism by modulating short-chain fatty acid and bile acid metabolism [14], all of which contribute to the amelioration of NAFLD.

Therefore, it is crucial to study the role of FXR and its signaling pathway in liver bile acid metabolism for exploring the pathogenesis of NAFLD and finding effective therapeutic targets. This study is aimed at studying NAFLD bile acid metabolism changes and FXR signaling pathway, exploring the effect of probiotics on the pathway, and seeking new therapy for NAFLD to provide a theoretical and experimental basis.

## 2. Materials and Methods

### 2.1. Animals

24 male Sprague-Dawley rats (160-180 g) were purchased from Shanghai Sino-British SIPPR/BK Laboratory Animal Co. Ltd. (Shanghai, China). All rats were housed in specific pathogen-free conditions (22°C, a 12 h light/dark cycle) with ad libitum access to standard laboratory chow. All animal experiments were approved by the ethics committee of Zhejiang Chinese Medical University (no. ZSLL-2018-048), and the study was conducted following the guidelines of the NIH *Guide for the Care and Use of Laboratory Animals*.

### 2.2. Animal Experimental Procedures

24 male SD rats were randomly divided into four groups (*n* = 6): a normal diet control (NC) group, a high-fat diet-fed (HFD) group, a high-fat diet-fed+probiotic treatment (HFD-P) group, and a high-fat diet-fed+atorvastatin (HFD-A) treatment. Atorvastatin has been proven to improve dyslipidemia in patients with NAFLD and improve NAFLD effectively. Thus, the atorvastatin treatment group was increased and compared with the probiotic treatment group to clearly show the effect of gut microbiota in NAFLD treatment. The rats were fed either a normal diet (10% kcal% fat LAD0011) or HFD (45 kcal% fat TP23000) (Trophic Animal Feed High-tech Co., Ltd, Nantong, China). After 6 weeks, rats in the NC and HFD groups were given normal saline. The HFD+P group rats were given 312 mg/kg/d probiotics by gavage once a day for 8 weeks (Compound Eosinophil-Lactobacillus Tablets, Tonghua Golden-Horse Pharmaceutical Industry Co., Ltd, Jilin, China; 10^7^ Eosinophil-Lactobacillus per gram). Rats in the HFD+A were given 10 mg/kg/d atorvastatin by gavage once a day (Lipitor, Pfizer, Dalian, China) for 8 weeks. The clinical dosage of Compound Eosinophil-Lactobacillus Tablets is two tablets at a time for adults (0.5 g each), three times a day. After conversion, the clinical dosage of adults is 50 mg/kg/d (the adult weight is considered 60 kg). The equivalent dose for rats is 6.25 times that of adults. Therefore, each rat is best given 312 mg/kg/d probiotics. In the same way, the dose of atorvastatin in rats is calculated to be 10 mg/kg/d. The rats were sacrificed at the end of 14 weeks, blood was taken from the abdominal vein under fasting anesthesia, and liver samples were taken.

### 2.3. Biochemical Analysis

Serological tests were used to detect the serum content of triglyceride (TG), cholesterol (CHOL), alanine aminotransferase (ALT), aspartate aminotransferase (AST), high-density lipoprotein (HDL), low-density lipoprotein (LDL), and total bile acid (TBA) (Nanjing Jiancheng Bioengineering Institute, Nanjing, China). The instrument is an automatic biochemistry analyzer (HITACHI, Japan).

### 2.4. Liver Histological Examination

The liver tissue of rats was fixed in 4% neutral-buffered formaldehyde for 24 hours, and then, hematoxylin-eosin (HE) staining was used to observe the presence of fat droplets in the liver under the microscope (Zeiss, Axio Scan Z1, Germany). The NAFLD activity score is regarded as a semiquantitative assessment of the degree of liver inflammation. NAS is calculated from the weighted sum of hepatocyte steatosis (0 to 3), lobular inflammation (0 to 3), and ballooning (0 to 2). According to the NAS, NAFLD is divided into “non-NASH” (NAS < 3), “edge NASH” (NASH = 3-4), and “definite NASH” (NAS = 5-8).

### 2.5. Detection and Analysis of Gut Microbiota

DNA from different faecal samples was extracted using a E.Z.N.A.® Stool DNA Kit (OMEGA Bio-Tek Inc., GA, USA) according to the manufacturer's instructions. The total DNA was eluted in 50 *μ*L of elution buffer and stored at -80°C until measurement in the PCR. The V3-V4 hypervariable region of the 16S rDNA gene uses primers 341F (5′-CCTACGGGNGGCWGCAG-3′) and 805R (5′-GACTACHVGGGTATCTAATCC-3′). The amplification was carried out as follows: initial denaturation at 98°C for 30 s, followed by 30 cycles of denaturation at 98°C for 10 s, annealing at 54°C for 30 s, elongation at 72°C for 45 s, and finally 72°C for 10 min. For each faecal sample, sequencing and bioinformatics were carried out by LC-Bio Technology Co., Ltd (Hangzhou, China) on the NovaSeq PE250 platform.

### 2.6. Real-Time qRT-PCR Analysis

Total RNA was isolated from the liver using the TaKaRa MiniBEST Universal RNA Extraction Kit (TaKaRa, Japan; cat. no. 9767) according to the manufacturer's instructions. Real-time PCR was performed using the TB Green™ Premix Ex Taq™ Kit (TaKaRa, Japan; Cat. no. RR820A) and a CFX384 Real-Time PCR system (Bio-Rad, USA). The PCR program included 1 cycle of 95°C for 3 min and 40 cycles of 95°C for 5 s and 60°C for 45 s. The specific primers used for amplification are shown in Table 1. The results were expressed by calculating the ^2-*ΔΔ*^CT values, and the housekeeping gene is *β*-actin.

### 2.7. Western Blotting Analysis

The sample preparation and extraction were carried out according to the standard scheme. Total proteins were extracted from liver tissues of rats using a total protein extraction kit (KeyGENBioTECH, KGP2100, Jiangsu, China). The protein content was determined by the BCA protein assay kit (MultiSciences, 70-PQ0011, Hangzhou, China). Then, we use SDS-PAGE gel (10%) isolate proteins, and they were transferred to PVDF membranes (Bio-Rad, USA). They were blocked with 5% skim milk in TBS-Tween 20 (TBST) for 1 h. The membranes were then incubated with primary antibodies overnight at 4°C. The primary antibodies are FXR monoclonal antibody, 1 : 1000 dilution (abs122163, Abisin); FGF15 monoclonal antibody, 1 : 1000 dilution (sc-398338, Santa Cruz); and *β*-actin monoclonal antibody, 1 : 5000 dilution (Multi Science Biotech, Cat. no. ab008). Then, the blots were incubated with the secondary antibodies HRP-conjugated goat anti-rabbit IgG (1 : 5000) dilution (Multi Science Biotech, Cat. no. GAR0072) or HRP-conjugated goat anti-mouse IgG (1 : 5000) dilution (Multi Science Biotech, Cat. no. GAM0072) for 1 h. Finally, protein expressions were detected with the enhanced chemiluminescence (ECL) method, and signals were captured with the Odyssey Fc (LI-COR, USA).

### 2.8. Statistical Analysis

The SPSS 26.0 software was used for statistical analysis. All quantitative data are presented as the means ± standard deviation. The significant differences between and within the different groups were examined using one-way ANOVAs, followed by Dunnett's test. Microbiome-related analysis figures were created by R software. *P* < 0.05 was considered statistically significant.

## 3. Results

### 3.1. Body Weight

**Figure 1:**
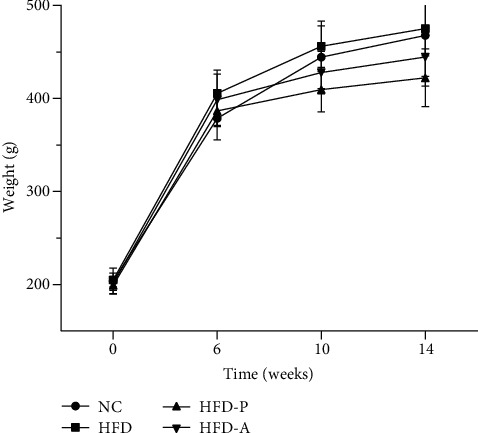
Changes in body weight.

In the 6^th^ week, compared with the NC group (378.7 ± 23.1, mean ± SD), the weight of the rats fed a high-fat diet increased, but the difference was not statistically significant (*P* > 0.05) (HFD: 405.5 ± 25.0, HFD-P: 386.7 ± 16.8, HFD-A: 398.7 ± 27.6, mean ± SD). At the end of the 10^th^ week and 14^th^ week, compared with the HFD group, the weight of the HFD-P group and HFD-A group decreased, but the difference was not statistically significant (*P* > 0.05) (Figure 1) (NC: 444.5 ± 33.7, HFD: 456.2 ± 27.1, HFD-P: 409.6 ± 23.9, HFD-A: 428 ± 22.9, 10^th^ week; NC: 467.8 ± 44.1, HFD: 475.2 ± 29.3, HFD-P: 422.3 ± 31.1, HFD-A: 444.7 ± 31.3, 14^th^ week, mean ± SD).

### 3.2. Histology Results

**Figure 2:**
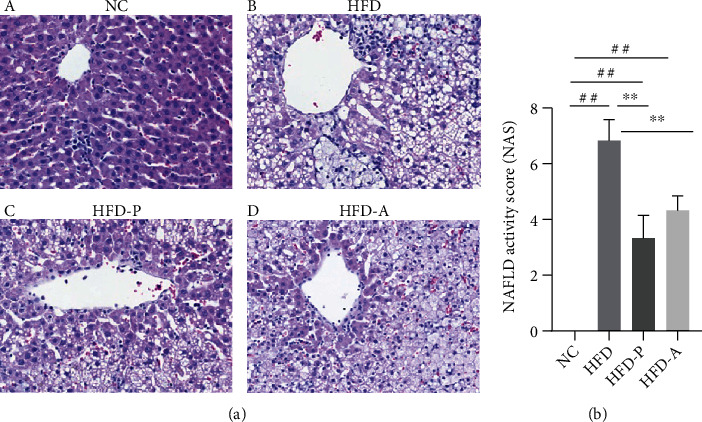
Effect of probiotics on the histology of liver tissue induced by HFD in NAFLD rats. (a) HE staining results: (A) NC group; (B) HFD group; (C) HFD-P group; (D) HFD-A group (×200 magnification). (b) NAFLD activity score results. ^##^*P* < 0.01 versus the NC group; ^∗∗^*P* < 0.01 versus the HFD group.

HE staining showed that in the NC group, the structure of hepatic lobules was clear and complete, without lipid infiltration. In the HFD group, there were evident steatosis, fatty vacuoles, disorganized structure of hepatic cord, and infiltration of inflammatory cells. However, the liver's fatty degeneration and inflammatory cell infiltration in the HFD-P and HFD-A groups were significantly reduced. The results of NAS also showed that the hepatic inflammation in the HFD group was significantly worse than that in the NC group. In addition, hepatic inflammation was greatly improved after treatment with probiotics (HFD-P group) and atorvastatin (HFD-A group) (Figure 2).

### 3.3. Biochemical Indexes

**Figure 3:**
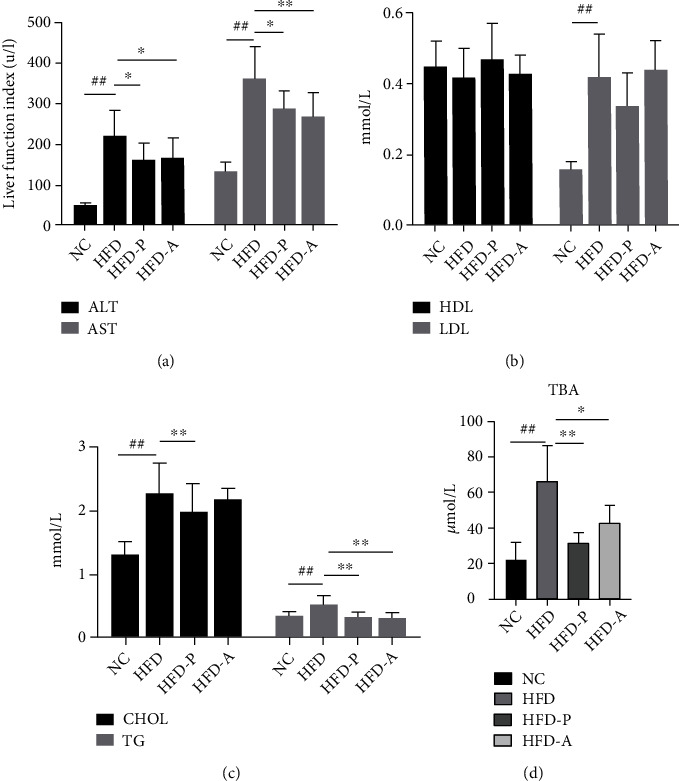
Biochemical index changes in all groups: (a) the levels of ALT and AST; (b) the levels of HDL and LDL; (c) the levels of CHOL and TG; (d) the level of TBA. ^#^*P* < 0.05 and ^##^*P* < 0.01 versus the NC group; ^∗^*P* < 0.05 and ^∗∗^*P* < 0.01 versus the HFD group.

Compared with the NC group, the levels of ALT, AST, TG, CHOL, LDL, and TBA in the HFD group increased significantly (*P* < 0.01), while the level of HDL decreased, but there was no statistical difference (*P* > 0.05); compared with the HFD group, the levels of ALT, AST, TG, and TBA in the HFD-P group and HFD-A group decreased (*P* < 0.05 or *P* < 0.01), and the level of CHOL in the HFD-P group decreased (*P* < 0.01) (Table 2, Figure 3).

### 3.4. Probiotics Improve Gut Microbiota in HFD-Induced NAFLD

**Figure 4:**
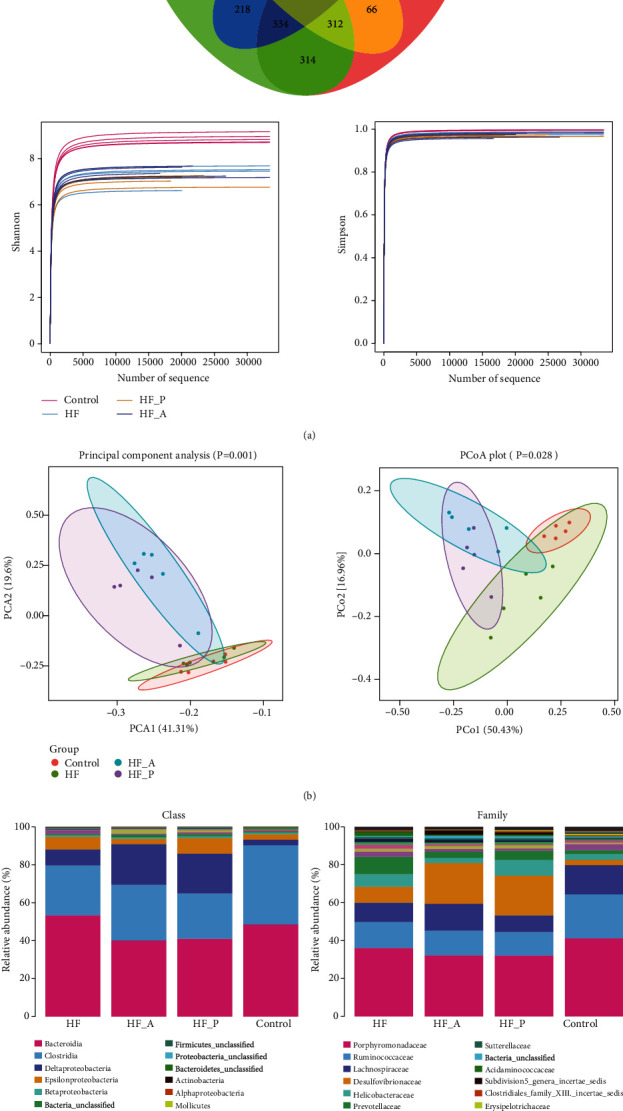
Probiotics improve gut microbiota in HFD-induced NAFLD: (a) Venn diagram, Shannon, and Simpson; (b) PCA and PCoA; (c) relative abundance of four groups in class and family level.

Compared with the NC group, the alpha diversity index (Shannon and Simpson) of the HFD group decreased significantly (*P* < 0.01, *P* < 0.05). The HFD-P group and the HFD-A group were upregulated considerably, and the bacterial diversity was increased (*P* < 0.05) (Figure 4(a)). The differences of gut microbiota among the four groups of rats can be classified by Principal Component Analysis (PCA) and Principal Coordinate Analysis (PCoA) (Figure 4(b)). The four groups can cluster on the PCoA map, and there is no overlap, indicating that there are differences in beta diversity of gut microbiota among the four groups of rats. The separation between the four groups was far, which represents that the extent of similarity between different microbial communities is low. The HFD-P group was close to the NC group after the intervention, which stated that probiotic intervention had a certain effect on the diversity of gut microbiota in NAFLD model rats. Compared with the NC group, *Bacteroidia* was increased and *Clostridia* was decreased in the HFD group at the class level. After the intervention, *Bacteroidia* was decreased in the HFD-P group and *Clostridia* was increased in the HFD-A group. At the family level, *Porphyromonadaceae* was decreased in the HFD group, the HFD-P group, and the HFD-A group, while *Desulfovibrionaceae was* increased in the HFD-P group and the HFD-A groups (Figure 4(c)). These results indicate that probiotics and atorvastatin can upregulate the diversity of gut microbiota and downregulate the abundance of pathogenic bacteria in NAFLD model rats, improving the imbalance of gut microbiota.

### 3.5. Effects of Probiotics on the Expression of FXR/FGF15 in the Liver of NAFLD Rats

**Figure 5:**
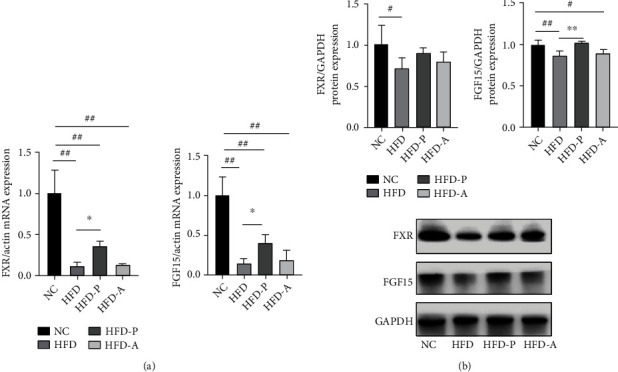
Effects of probiotics on the expression of FXR/FGF15 in the liver of NAFLD rats: (a) the expression of FXR and FGF15 in the liver of NAFLD rats; (b) Western blot for FXR and FGF15 in the liver of NAFLD rats. ^#^*P* < 0.05 and ^##^*P* < 0.011 versus the NC group; ^∗^*P* < 0.05 and ^∗∗^*P* < 0.01 versus the HFD group.

The expression of FXR and FGF15 mRNA in liver tissue of the HFD group was significantly lower than that of the NC group (*P* < 0.01), and after probiotic intervention, the expression of FXR and FGF15 mRNA was increased dramatically than the HFD group (*P* < 0.05). There was no significant difference between the HFD-A group and the HFD group after atorvastatin intervention (*P* > 0.05). The protein expression of FXR and FGF15 in liver tissue of the HFD group was significantly lower than that of the NC group (*P* < 0.05, *P* < 0.01). After probiotic intervention, FGF15 was markedly higher than that of the HFD group (*P* < 0.01), and there was no significant difference in FXR (*P* > 0.05). There was no significant difference between the HFD-A group and the HFD group (*P* > 0.05) (Figure 5).

## 4. Discussion

Emerging evidence has suggested that bile acid metabolism is closely associated with NAFLD [15, 16]. Bile acids are important signaling molecules that participate in glycolipid metabolism and energy metabolism and modulate inflammation in enterohepatic circulation and peripheral organs [17, 18].

Some studies have shown that high-fat diet (HFD) can change the composition of gut microbiota resulting in loss of commensal bacteria, leading to low-grade inflammation (LGI) and NAFLD [19, 20]. The composition of the bile acid (BA) pool is modified by gut microbiota. Perturbations of gut microbiota shape the BA composition, which, in turn, may alter essential BA signaling and affect host metabolism [21]. Bile acids are endogenous ligands, which can activate nuclear receptors, such as farnesoid X receptor (FXR). In the liver, FXR regulates cholesterol metabolism by regulating the expression of cholesterol 7*α*-hydroxylase (CYP7A1). In intestinal epithelial cells, activated FXR can induce the synthesis of fibroblast growth factor 15/19 (rat/human), which inhibits the expression of CYP7A1 to limit the synthesis of bile acids [22].

Clinical and animal experiments have proved that probiotics can improve the imbalance of gut microbiota and intestinal inflammation [23, 24]. Many types of probiotics were studied for NAFLD treatment; the most common include *Lactobacillus* and *Bifidobacteria.* The mechanism mainly includes improving transaminase, liver steatosis, reducing liver inflammation, and regulating gut microbiota [25]. This study is aimed at targeting probiotic (Eosinophil-Lactobacillus) intervention to regulate the gut microbiota-FXR-FGF15 axis and improve HFD-induced NAFLD in rat. This work will provide experimental basis for probiotic monotherapy or combination therapy in the treatment of NAFLD.

In this study, a NAFLD rat model was established by feeding rats with high-fat diet for 6 weeks. Compared with the NC group, the serum levels of ALT, AST, TG, CHOL, and LDL were increased, and HDL in the HFD group was decreased. The content of TBA in serum increased significantly. Meanwhile, HE staining has shown that the structure of hepatic lobules was clear and complete, without lipid infiltration in the NC group. In the HFD group, there were obvious steatosis, fatty vacuoles, disorganized structure of hepatic cord, and infiltration of inflammatory cells. These findings indicated that the lipid metabolism and bile acid metabolism were disordered, and the NAFLD model was successfully established. Compared with the HFD group, the levels of ALT, AST, TG, CHOL, and TBA in the HFD-P group and HFD-A group were lower. The levels of LDL were lower, and the levels of HDL were higher. However, there was no statistical difference, suggesting that probiotics and statins have a particular role in protecting the liver and regulating lipid and bile acid metabolism.

Gut microbiota is closely related to bile acid metabolism. The main pathway to regulate bile acid metabolism is hydrolysis combined with bile acid, which makes free bile acid dehydroxylation and complete modification [26]. In this study, we analyzed the changes of gut microbiota in four groups. At the phylum level, Bacteroidia was increased, and Clostridia was decreased in the HFD group. The phylum of Bacteroidia was reduced in the HFD-P group and raised in the HFD-A group after intervention. At the family level, Porphyromonadaceae was decreased in the HFD group, the HFD-P group, and the HFD-A group, while Desulfovibrionaceae was increased in the HFD-P group and the HFD-A group. These results indicate that probiotics and atorvastatin can upregulate gut microbiota diversity and downregulate the abundance of pathogenic bacteria in NAFLD model rats, improving gut microbiota dysbiosis.

FXR/FGF-15 is an adverse feedback regulation pathway of bile acid synthesis; FXR agonist regulated faecal bile acid levels in probiotic-treated mice [27]. In our report, the mRNA and protein expressions of FXR and FGF15 in liver tissues in the HFD model group were significantly lower than those in the NC group. After probiotic treatment, the mRNA and/or protein expressions of FXR and FGF15 were substantially higher than those in the HFD group, indicating that probiotics may affect bile acid metabolism by upregulating the expression of the FXR/FGF15 pathway. The improvement effect of atorvastatin was not noticeable.

## 5. Conclusion

In conclusion, our study demonstrated that probiotics had a protective effect against NAFLD in a rat model; its treatment significantly ameliorated the liver pathology injuries and serum lipid profiles and alleviated hepatic steatosis in HFD diet-fed rats; probiotics may affect bile acid metabolism by upregulating the expression of the FXR/FGF15 pathway and improving the gut microbiota dysbiosis. In addition, these protective mechanisms of probiotics on NAFLD may be related to a reduction in blood lipids, improved liver pathology, and increased bile acid receptor expression via the gut microbiota/FXR/FGF15 signaling pathway.

## Figures and Tables

**Table 1:** The specific primers used for amplification.

Name	Primers (5′⟶3′)		NCBI gene ID
*β*-Actin	Sense	TGCTGTCACCTTCACCGTTC	81822
	Antisense	GTCCACCGCAAATGCTTCTA	

FXR	Sense	CTCCCTGCATGACTTTGTTGTC	60351
	Antisense	AAGAGATGGGAATGTTGGCTG	

FGF15	Sense	AAGTGGAGTGGGCGTATTGT	170582
	Antisense	AGTGGACCTTCATCCGACAC	

**Table 2:** Biochemical indexes in all groups.

	NC	HFD	HFD-P	HFD-A
ALT (U/L)	50.4 ± 4.2	221.6 ± 60.8^##^	161.8 ± 40.5^∗^	167.0 ± 48.1^∗^
AST (U/L)	133.4 ± 20.7	362.8 ± 75.4^##^	288.6 ± 42.0^∗^	267.8 ± 57.4^∗∗^
HDL (mmol/L)	0.45 ± 0.07	0.42 ± 0.08	0.47 ± 0.10	0.43 ± 0.05
LDL (mmol/L)	0.16 ± 0.02	0.42 ± 0.12^##^	0.34 ± 0.09	0.44 ± 0.08
CHOL (mmol/L)	1.32 ± 0.19	2.27 ± 0.47^##^	1.98 ± 0.44^∗∗^	2.18 ± 0.17
TG (mmol/L)	0.35 ± 0.06	0.53 ± 0.13^##^	0.33 ± 0.07^∗∗^	0.31 ± 0.08^∗∗^
TBA (*μ*mol/L)	21.85 ± 10.07	66.28 ± 19.9^##^	31.42 ± 6.04^∗∗^	42.67 ± 9.88^∗^

^##^*P* < 0.01 and ^#^*P* < 0.05 versus NC; ^∗∗^*P* < 0.01 and ^∗^*P* < 0.05 versus HFD. *n* = 6 in each group.

## Data Availability

The data in this study is available from the corresponding author upon reasonable request.

## Abbreviations

ALT: Alanine aminotransferase
AST: Aspartate aminotransferase
CHOL: Cholesterol
CYP7A1: Cholesterol 7*α*-hydroxylase
FGF15: Fibroblast growth factor 15
FXR: Farnesoid X receptor
HDL: High-density lipoprotein
HE: Hematoxylin-eosin
HFD: High-fat diet
LDL: Low-density lipoprotein
LGI: Low-grade inflammation
NAFLD: Nonalcoholic fatty liver disease
TBA: Total bile acid
TG: Triglyceride
PCA: Principal Component Analysis
PCoA: Principal Coordinate Analysis.
